# Purification of Antibodies From Human Milk and Infant Digestates for Viral Inhibition Assays

**DOI:** 10.3389/fnut.2020.00136

**Published:** 2020-08-25

**Authors:** Baidya Nath P. Sah, Jiraporn Lueangsakulthai, Benjamin R. Hauser, Veronique Demers-Mathieu, Brian Scottoline, Manoj K. Pastey, David C. Dallas

**Affiliations:** ^1^Nutrition Program, School of Biological and Population Health Sciences, College of Public Health and Human Sciences, Oregon State University, Corvallis, OR, United States; ^2^Division of Neonatology, Department of Pediatrics, School of Medicine, Oregon Health & Science University, Portland, OR, United States; ^3^Carlson College of Veterinary Medicine, Oregon State University, Corvallis, OR, United States

**Keywords:** infant digestion, human milk, recombinant IgG1κ antibody, palivizumab, extraction, respiratory syncytial virus (RSV), ELISA, RSV neutralization assay

## Abstract

Oral administration of enteric pathogen-specific immunoglobulins may be an ideal approach for preventing infectious diarrhea in infants and children. For oral administration to be effective, antibodies must survive functionally intact within the highly proteolytic digestive tract. As an initial step toward assessing the viability of this approach, we examined the survival of palivizumab, a recombinant monoclonal antibody (IgG1κ), across infant digestion and its ability to neutralize respiratory syncytial virus (RSV). Human milk and infant digestive samples contain substances known to interfere with the RSV neutralization assay (our selected functional test for antibody survival through digestion), therefore, antibody extraction from the matrix was required prior to performing the assay. The efficacy of various approaches for palivizumab purification from human milk, infant's gastric and intestinal digestates, including casein precipitation, salting out, molecular weight cut-off, and affinity chromatography (protein A and G) were compared. Affinity chromatography using protein G with high-salt elution followed by 30-kDa molecular weight cut-off centrifugal filtration was the most effective technique for purifying palivizumab from human milk and infant digestates with a high yield and reduced background interference for the viral neutralization assay. This work is broadly applicable to the optimal isolation of antibodies from human milk and infant digesta for viral neutralization assays, enables the examination of how digestion affects the viral neutralization capacity of antibodies within milk and digestive samples, and paves the way for assessment of the viability of oral administration of recombinant antibodies as a therapeutic approach to prevent enteric pathogen-induced infectious diarrhea in infants.

## 1. Introduction

Enteric pathogen-induced infectious diarrhea is one of the leading causes of death in children in developing countries (1). One potential approach to preventing enteric pathogen-induced diarrhea in infants is oral administration of recombinant, pathogen-specific immunoglobulins. Such enteric pathogen-specific antibodies would have to survive functionally intact across the infant digestive tract to provide clinical benefit. The infant digestive system contains various proteolytic enzymes and a broad range of pH from 3.5 to 8 (2, 3) that could degrade recombinant antibodies. To determine the feasibility of this approach, we examined the *in vivo* survival of a recombinant antibody within the infant digestive system.

As a proxy for enteric pathogen-specific recombinant antibodies, the functional survival of orally delivered palivizumab, the recombinant monoclonal antibody (IgG1κ) against respiratory syncytial virus (RSV), was examined. This antibody has been approved by the FDA to provide passive immunity against infection by RSV in infants via intramuscular injection. Knowledge of the extent to which palivizumab maintains its RSV-neutralizing function across the infant digestive system requires the isolation of the immunoglobulin, as the complex matrices of milk, and infant's gastric and intestinal digestates have a variety of components, including proteases, protease inhibitors, immunoglobulins (SIgA, IgG, and IgM), β-casein, lactoferrin and lactoperoxidase, milk fat, cells and bacteria that can interfere with the RSV neutralization assay (4–7).

An optimal method for antibody purification and removal of interfering substances from human milk and infant digestive samples for an RSV neutralization assay has not been determined. The aim of this study was to establish an optimal antibody purification method that allows high retention of palivizumab while removing substances from human milk and infant digestive samples that interfere with the neutralization assay. Establishing such a method provides a means to evaluate the feasibility of oral delivery of enteric-pathogen specific antibodies in the prevention of infectious diarrhea.

## 2. Materials and Methods

### 2.1. Digestion of Human Milk

Test samples for the experiments described herein included pooled donor human milk with or without palivizumab exposed to simulated infant gastrointestinal conditions (*in vitro* digestion), and source human milk, gastric digestate and intestinal digestate collected after infants were fed mother's milk with or without palivizumab (*in vivo* digestion).

#### 2.1.1. *In vitro* Digestion

Pooled donor human milk without and with palivizumab was subjected to *in vitro* infant simulated digestion as described by Nguyen et al. (8) with some modifications. The average protein content of human milk was reported as 10 mg/mL in our previous study (9), and this value was used to calculate the amount of pepsin and pancreatin to add to samples for *in vitro* infant digestion. To simulate infant gastric fluid for testing digestion *in vitro*, pepsin from porcine gastric mucosa (powder ≥ 400 units/mg protein, MilliporeSigma) was dissolved in 0.15 M NaCl solution, pH 4.0, and 250 μL of this solution was added to 500 μL of human milk to achieve 22.75 U protease activity per mg protein.

The mixture was adjusted to pH 4.0 ± 0.05 using 0.1 N HCl (~80 μL) and incubated for 1 h in an Eppendorf ThermoMixer® C (Eppendorf AG, Hamburg, Germany) at 37°C with shaking at 300 rpm. For *in vitro* gastrointestinal digestion, the simulated gastric digestate (830 μL) was further mixed with 830 μL of simulated intestinal fluid, which was prepared by dissolving pancreatin from porcine pancreas (8 × USP, MilliporeSigma) in 0.15 M NaCl containing 2 mM bile salt solution (pH 8.0) to achieve 3.5 U protease activity per mg sample protein. The mixture was adjusted to pH 8.0 ± 0.05 with 0.1 N NaOH (~90 μL) and incubated for 2 h at 37°C with shaking at 300 rpm. Final volumes of all samples were adjusted to 1.75 mL by adding phosphate-buffered saline (PBS). Aliquots of 350 μL were pipetted into Protein Lobind tubes (Eppendorf AG, Hamburg, Germany) and stored immediately at −80°C to prevent further enzymatic reactions.

#### 2.1.2. *In vivo* Digestion

Human milk and infant gastric and intestinal samples were collected as described in our previous publication (10).

### 2.2. Approaches to Remove Interfering Substances From Human Milk and Infant Digestates

Purification approaches, such as centrifugation, 0.2-μm filtration, MWCO membrane filtration, precipitation of immunoglobulin through salting-out with (NH_4_)_2_SO_4_, precipitation of casein with HCl, dialysis and affinity chromatography (protein A and G), were tested in various combinations in the following Experiments (2.2.1–2.2.9) to recover palivizumab and remove interfering substances from milk and infant digestates prior to the viral inhibition assay. Our criteria were that the final approach needed to have >50% extraction efficiency and to eliminate background milk inhibition sufficient to detect the contribution of palivizumab to RSV neutralization in our functional assay.

#### 2.2.1. Centrifugation (11,292 g) and 0.2-μm Filtration

Palivizumab was added to pooled donor human milk and an infant's gastric content at 200 μg/mL. One milliliter of each sample was centrifuged twice for 10 min at 11,292 *g*, 4°C, and the infranate (liquid portion between the upper lipid and lower particulate) was collected and filtered through a sterile polyethersulfone (PES) filter (0.2 μm) in a biosafety cabinet. The same sample types with no palivizumab added were also processed with the same conditions for the neutralization assay.

#### 2.2.2. Centrifugation and 100-kDa MWCO

Palivizumab was added to pooled donor human milk, an infant's gastric content and simulated intestinal digestate (*in vitro* digestion of pooled donor human milk) at 100 μg/mL and 1 mL of each sample was centrifuged two times for 10 min at 4,000 *g*, 4°C. Supernatants (0.5 mL) were collected, transferred to a 100-kDa MWCO centrifugal filtration device (Amicon Ultra-0.5 Centrifugal Filter Unit, MilliporeSigma, St. Louis, MO) and centrifuged at 14,000 *g*, 4°C for 10 min. The retentate was collected and filtered through a sterile PES filter (0.2 μm) in a biosafety cabinet. The same sample types with no palivizumab added were also processed with the same conditions for the neutralization assay.

#### 2.2.3. Ammonium Sulfate Precipitation (Salting Out)

Five hundred microliters of pooled donor human milk containing 100 μg/mL palivizumab were mixed with 500 μL of saturated ammonium sulfate solution (100% saturated solution at RT) to salt out immunoglobulins, including palivizumab. The mixture was incubated overnight at 4°C with agitation at 300 rpm and centrifuged for 40 min at 14,000 *g*, 4°C. The supernatant (500 μL) was decanted. The pellet was resuspended by pipetting and depipeting in the remaining supernatant, and diluted in PBS (pH 7.4) containing 0.05% Tween-20 (PBST) (Bio-Rad, Richmond, CA) and 10% human AB serum (Corning, Manassas, VA) for palivizumab determination.

#### 2.2.4. Skimming Fat, Casein Precipitation, 0.2-μm Filtration and 100-kDa MWCO

Five hundred microliters of pooled donor human milk with 100 μg/mL palivizumab was skimmed by centrifugation (4,000 *g*, 4°C for 20 min). Casein was precipitated from the skim milk by adding 0.1 N HCl dropwise to achieve pH 4.5 ± 0.05 and centrifuging for 20 min at 4,000 *g*, 4°C. After centrifugation, the supernatant was filtered using a 0.2-μm PES syringe filter (as described in Experiment 2.2.1) to remove small, coagulated casein particles followed by 100-kDa MWCO filtration for 3 min (as described in Experiment 2.2.2) at 14,000 *g*, 4°C. Prior to use, the 100 kDa-MWCO membrane was cleansed by centrifuging and passing through 500 μL of 0.1 M NaOH twice followed by 500 μL of nanopure water twice. The final volume was adjusted to 2 mL using nanopure water.

#### 2.2.5. Protein A (Gravity-Flow Method)

Palivizumab was added to pooled donor human milk and an infant's gastric content at 100 μg/mL for purification with protein A columns (Pierce™ Protein A IgG Purification Kit, 5 mL; Thermo Fisher Scientific, Waltham, MA). The protein A columns and buffers were allowed to come to RT. The storage solution was discarded from the column and 5 mL of Protein A IgG binding buffer (Thermo Fisher Scientific) was added to the column and allowed to flow through to equilibrate the column. One hundred microliters of the samples were diluted with 100 μL of the binding buffer, poured into the column and allowed to drain from the resin portion of the column. The column was washed with 15 mL of the binding buffer to remove non-specifically bound non-IgG components. IgG elution Buffer (10 mL) (0.1 M glycine; pH 2–3) was added into the column. The eluate was collected in 1 mL fractions and 50 μL of neutralization buffer (1 M Tris-HCl; pH 8.0) was added to each 1 mL fraction to neutralize the eluates. Absorbance of each fraction was measured at 280 nm using a NanoDrop spectrophotometer (Thermo Scientific, Wilmington, DE) and fractions having a high absorbance were combined.

#### 2.2.6. Protein G Spin Plate (Low-pH Elution)

Pooled donor human milk with 200 μg/mL palivizumab, and simulated gastric and intestinal digestates of that sample were subjected to purification using Protein G Spin Plates for IgG Screening (Thermo Fisher Scientific). The spin plate and all buffers were equilibrated to RT (30 min). Two hundred microliters of binding buffer (100 mM sodium phosphate, 150 mM sodium chloride; pH 7.2) were added to each well to equilibrate. The plate was centrifuged for 1 min at 1,000 *g*, 20°C. The flow-through was discarded. This washing step was repeated once. Samples (400 μL) were diluted 1:2 (v/v) in the binding buffer and added to the wells. Dilution of the samples in the binding buffer was required to maintain appropriate ionic strength and optimal binding pH. An aliquot of the diluted sample (400 μL) was loaded into a well of the plate. The plate was rocked for 30 min on a rocking plate at 100 rpm. The plate assembly was centrifuged as described above. The flow-through was reloaded, rocked and centrifuged to recover any palivizumab lost in the flow-through. The remaining diluted sample was passed through the resin in the same well as described above. The resin in the well was washed with 400 μL of binding buffer four times to remove non-specifically bound contaminating proteins. Seventeen microliters of neutralization buffer (1 M Tris-HCl; pH 8.0) were added to each well of the collection plate and the purification plate was placed on the collection plate. Two hundred microliters of elution buffer (0.1 M glycine; pH 2–3) were added to each well, rocked for 1 min and centrifuged for 1 min at 1,000 *g* and 20°C. These elution steps were repeated for a total of three times and the three eluates were combined. The same sample types with no palivizumab added were also processed with the same conditions used for the neutralization assay.

#### 2.2.7. Protein G Spin Plate (Low-pH Elution) and 100 kDa-MWCO

Protein G extracts from Experiment 2.2.6 were further purified using 100-kDa MWCO as described in Experiment 2.2.4 to remove non-specifically bound proteins, salts and other contaminants. The elution buffer was exchanged with nanopure water and the retentate was collected.

#### 2.2.8. Protein G Spin Plate (Low-pH Elution) and 100-kDa Dialyzer

Palivizumab was added to pooled donor human milk at 100 μg/mL and subjected to *in vitro* simulated infant digestion as described in section Centrifugation (11,292 g) and 0.2-μm Filtration. Palivizumab was extracted from 1.5 mL of the human milk and *in vitro* simulated infant digestates using the protein G spin plate as described in the Experiment 2.2.6. A dialyzer (Micro Float-A-Lyzer device; 100-kDa MWCO; Spectrum Laboratories, Inc., Rancho Dominguez, CA) was used to remove interfering substances from the protein G extract. The dialyzer was soaked first in 10% ethanol followed by thoroughly flushing and soaking in nanopure water to remove glycerin and achieve maximum membrane permeability. Sample (500 μL) was loaded, using a 1-mL syringe, into the sample chamber of the device. The device was placed in the dialysate buffer (histidine buffer (25 mM histidine, 1.6 mM glycine; pH 6.0), which was the storage buffer of palivizumab supplied by MedImmune). The buffer was stirred during the entire dialysis period (18 h). The dialysis was performed at 4°C and the buffer was exchanged at 1, 2, and 15 h of dialysis. After completion of dialysis, the device was removed from the dialysate buffer and the sample was retrieved. After removing the sample, the empty sample chamber was loaded with 50 μL of histidine buffer and a small amount of air. The cap was replaced, and the device was shaken gently several times to rinse the membrane. The rinsed volume was retrieved and combined with the sample.

#### 2.2.9. Protein G Spin Column (High-Salt Elution) and 30 kDa-MWCO

**Figure 1:**
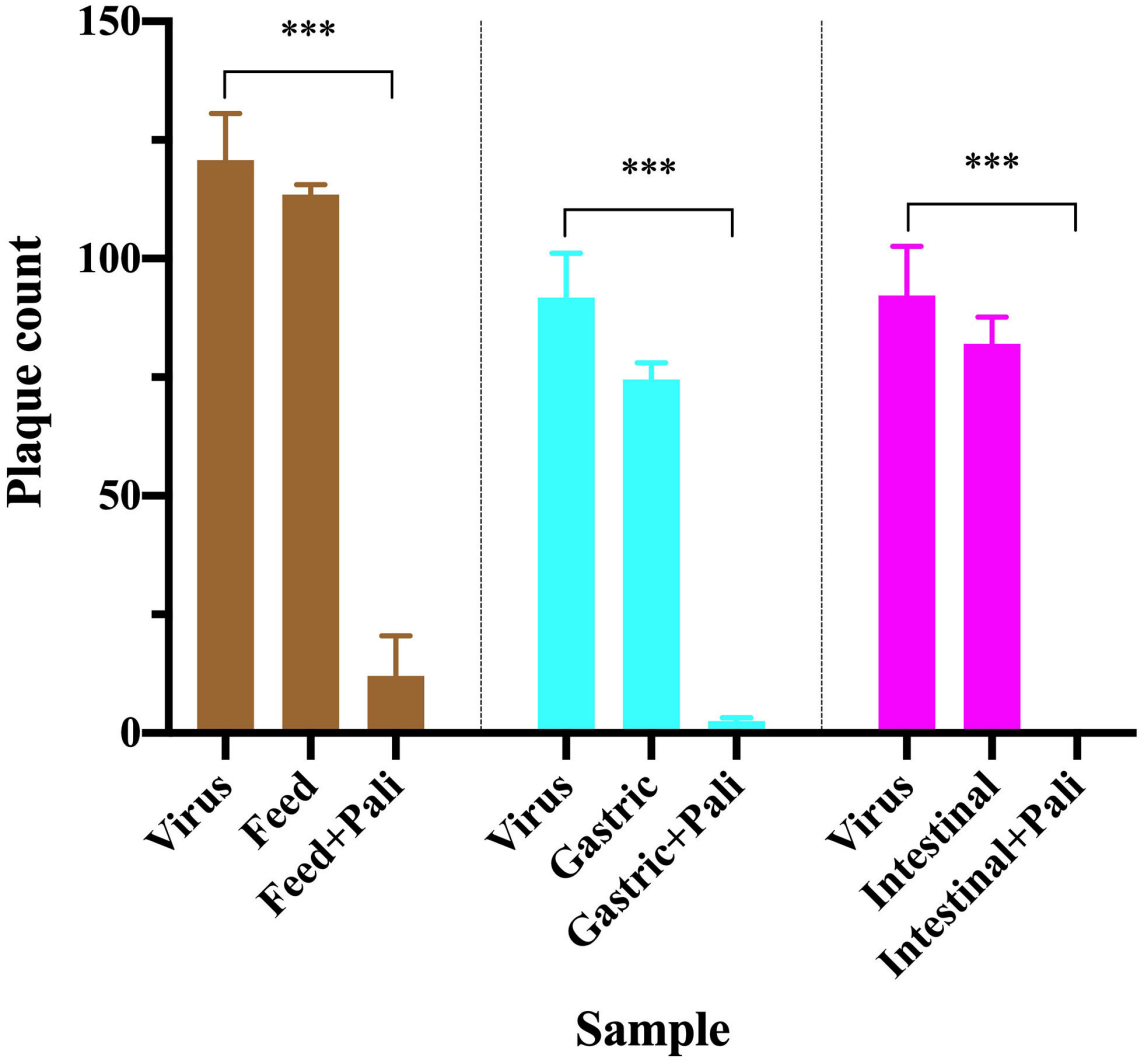
RSV plaque counts in virus only wells (positive control), wells with virus treated with purified samples of human milk, gastric and intestinal digestates from 1 infant fed without or with 60 μg/mL palivizumab. Values are mean ± SD, *n* = 4 wells for virus only and *n* = 2 wells for virus treated with each sample type. Data are shown at the dilution for insignificant background effect for each sample type (29.25-fold for feed, 20.22-fold for gastric, and 12.33-fold for intestinal samples). Asterisks show statistically significant differences (***P < 0.001) between positive control (virus only well) and feed (without or with palivizumab), gastric (without or with palivizumab) and intestinal (without or with palivizumab) within each sample type using unpaired *t*-tests.

An infant was fed mother's milk without or with palivizumab at 60 μg/mL, and gastric and intestinal digestates were collected as described in section *in vivo* Digestion. The extraction procedure was performed for both sets of samples with and without palivizumab. Palivizumab was purified from these samples using a protein G spin column (Thermo Fisher Scientific). The spin column and all buffers were equilibrated to RT (30 min). The column was centrifuged for 30 s at 5,000 g, 20°C to pass through the storage solution and equilibrated by adding 400 μL of the Pierce™ protein G IgG binding buffer (proprietary composition, pH 5.0, containing 0.02% sodium azide) to the column followed by centrifugation for 30 s at 5,000 g, 20°C. The equilibration step was repeated once. Samples were separately diluted with the binding buffer at least in the ratio of 1:3 (v/v) to ensure optimal ionic strength and pH for binding. The diluted sample was centrifuged for 10 min at 1,000 *g*, 4°C. The supernatant was collected for palivizumab extraction. The pellet was dissolved in 1 mL binding buffer, centrifuged as described above, and the supernatant was collected and combined with the previous supernatant. An aliquot of this supernatant prepared from sample-buffer mixture (500 μL) was added to the protein G spin column and mixed end-over-end for 10 min and centrifuging for 30 s at 5,000 *g*, 20°C. The column was washed by adding 500 μL of the binding buffer followed by mixing to resuspend the resin and centrifuging for 30 s at 5,000 g, 20°C. These wash steps were repeated 9 times. Palivizumab bound to the resin was eluted by adding 500 μL of the Pierce™ gentle Ag/Ab elution buffer (proprietary composition, high ionic strength, pH 6.6) to the column, mixing end-over-end and centrifuging for 60 s at 5,000 g, 20°C. The elution step was repeated a total of eight times. To remove remaining interfering substances, the protein G extract was added to a 30-kDa MWCO centrifugal filter unit. Prior to addition of the sample, 5 mL of Dulbecco's Modified Eagle Medium (DMEM), without fetal bovine serum (FBS), were added to the device followed by centrifugation for 3 min at 3,000 *g*, 4°C to wash the apparatus. This washing step was repeated once. Four milliliters of each protein G extract were combined with 5 mL of DMEM (no FBS, with antibiotic), added to the MWCO device and centrifuged the device for 10 min at 1,000 *g*, 4°C. To allow for additional removal of interfering substances, 5 mL of DMEM (no FBS, with antibiotic) were added to the MWCO device and the device was centrifuged as described above (repeated 2 times). The retentate was collected from the MWCO device into the Protein Lobind tubes. The same sample types with no palivizumab added were also processed with the same conditions for the neutralization assay (Table 1). To confirm whether this approach adequately removed interfering substances, we also compared the neutralization capacity of milk, gastric and intestinal samples without and with palivizumab (Figure 1).

**Table 1:** Dilution number of samples (human milk, gastric, and intestinal digestates) without palivizumab for insignificant effect of background on the neutralization assay (statistical evaluation method described in section Statistical Analysis).

**Sample**	**Sample dilution number for insignificant effect of background on neutralization activity**			
	**Unpurified**	**Experiment 2.2.2** **(centrifugation, 100-kDa MWCO and 0.2-μm filtration)**	**Experiment 2.2.6** **(Protein G spin plate)**	**Experiment 2.2.9** **(Protein G spin column and** **30-kDa MWCO)**
Whole milk	>28,672	>864	–	29.25
Gastric digestate	>17,268	–	>78	20.22
Intestinal digestate	>8,192	–	>36	12.33

*For the unpurified sample, Experiments 2.2.2 and 2.2.6, gastric and intestinal digestates were prepared by in vitro digestion of pooled donor human milk. For Experiment 2.2.9, gastric and intestinal samples were collected from an infant as described in Section Digestion of Human Milk. –: Not determined*.

### 2.3. Determination of Palivizumab Content Using Anti-idiotype ELISA

An anti-idiotype enzyme-linked immunosorbent assay (ELISA) was performed to determine palivizumab content in the samples by employing HCA261 (Bio-Rad) as a capture antibody and HCA262P (Bio-Rad) as a detection antibody according to the protocol established by Bio-Rad, described in our previous publication (10), with some modifications. Briefly, 100 μL of HCA261 at 1 μg/mL in PBS was dispensed in each well of a clear flat-bottom 96-well plate. After overnight incubation at 4°C, unbound HCA261 antibodies were washed out by washing wells three times with 150 μL of PBST and blocked unbound sites on the wells with 150 μL of PBST containing 5% bovine serum albumin for 1 h at RT. Samples were diluted 2-fold with PBST containing 10% human AB serum, and 2-fold serially diluted palivizumab standards were also prepared in PBST containing 10% human AB serum in the range from 10,000 to 5 ng/mL. The wells were washed three times with PBST and 100 μL of the palivizumab standards/diluted samples were added to each well and incubated for 1 h at RT. One hundred microliters of 2 μg/mL HCA262P in HISPEC assay diluent (Bio-Rad) were added to each well after washing three times with PBST. The microplate was incubated for 1 h at RT. The wells were rewashed three times with PBST and 100 μL of 3,3′,5,5′-tetramethylbenzidine substrate solution (Thermo Fisher Scientific) was added to each well. The microplate was incubated for 5 min at RT. After adding 50 μL of 2 N sulfuric acid to each well, absorbance of the reaction mixture in each well was measured at 450 nm using a microplate reader (Spectramax® M2, Molecular Devices, Sunnyvale, CA).

### 2.4. Determination of Cytotoxic Effect of Human Milk and Infant Digestates on HEp-2 Cells

The cytotoxic effect of samples on HEp-2 cells (ATCC® CCL23™; up to 25 passages) was assessed employing CellTiter 96® AQ_ueous_ One Solution Reagent (Promega Corp., Madison, WI) in a colorimetric assay (11). The CellTiter 96® AQ_ueous_ One Solution Reagent consisted of a tetrazolium compound [3-(4,5-dimethylthiazol-2-yl)- 5-(3-carboxymethoxyphenyl)-2-(4-sulfophenyl)-2H- tetrazolium, inner salt; MTS] and an electron-coupling reagent (phenazine ethosulfate). Dehydrogenase enzymes, which are only active in live cells, reduce MTS to a formazan product that exhibits an absorbance peak at 490 nm. Briefly, 100 μL of a logarithmically growing HEp-2 cell suspension in DMEM containing 10% FBS and 1% antibacterial-antimycotic solution (3.0 × 10^4^ cells/mL) were dispensed into each well of a 96-well flat-bottomed tissue culture plate, and the plate was incubated at 37°C for 24 h in a 5% CO_2_ incubator to allow cellular adherence. The medium was replaced with 100 μL of 2-fold serially-diluted samples in DMEM growth medium. The microplate was further incubated at 37°C for 24 h in the CO_2_ incubator. Twenty microliters of CellTiter 96® AQ_ueous_ One Solution Reagent were added to each well, incubated at 37°C for 2 h in the CO_2_ incubator, and absorbance at 490 nm was measured using the microplate reader. The plate also included blank wells containing the same volume of DMEM growth medium instead of sample. The cytotoxic effect of samples was calculated using Equation (1).

$Cytotoxicity,\;\%\;=\frac{A_{b}-A_{s}}{A_{b}}\;\times 100\;\cdot \cdot \cdot \;\left(1\right)$; where, A_s_ was the absorbance of sample; A_b_ was the absorbance of blank.

### 2.5. Determination of Plaque Reduction Neutralization Titer

Neutralization titer of samples against RSV was determined using plaque reduction neutralization assay as described in our previous publication (10). Dilution values were corrected for any dilution/concentration during sample extraction and included the 2 × dilution from mixing the sample 1:1 with virus suspension prior to applying to HEp-2 cell wells.

### 2.6. Statistical Analysis

To determine the dilution at which the effect of background interfering substances were removed from the plaque reduction neutralization assay, we tested for significant differences (at *p* < 0.05) between the plaque count at each dilution of samples without palivizumab compared with the plaque count of the control wells with virus without samples using unpaired *t*-tests. The lowest dilution at which there was no significant difference was defined as the dilution value for the “insignificant effect of background” as used in Table 1. For the final extraction technique, to determine whether the background effect was reduced enough to observe the neutralizing capacity of palivizumab in the sample, the plaque counts from the virus mixed with purified milk, gastric and intestinal samples without or with palivizumab were tested for significant differences compared with the virus only well (*p* < 0.05) using unpaired *t*-tests (Figure 1). GraphPad Prism software (version 8.2.1) was used for statistical analyses.

## 3. Results and Discussion

Substances present in milk and digestive samples are capable of interfering with the RSV neutralization assay and prevent accurate measurement of the neutralizing capacity specific to added palivizumab and its survival across digestion. Thus, this work aimed to extract the monoclonal antibody palivizumab from human milk and infant digestive samples to remove interfering substances from these samples prior to viral neutralization assays.

The intrinsic RSV neutralization activity of human milk and digestive samples was quantified to establish the extent to which they contributed to a background level of assay interference prior to the addition of palivizumab. Whole human milk and simulated infant digestates without palivizumab inhibited RSV infection of HEp-2 cells (a host cell for RSV) up to the maximum number of dilutions tested in the experiment (> 28,000 × dilution of milk; Table 1).

We also tested the extent to which the human milk and gastric samples were cytotoxic to HEp-2 cells. More than 10% inhibition remained at dilutions of >200 × for milk and >500 × for gastric digestates (Table 2). The data demonstrate that these samples were cytotoxic to HEp-2 cells and indicate the background interference of the samples on the neutralization assay is in part due to the direct cytotoxic effect of the samples on the HEp-2 cells.

**Table 2:** Dilution number at which samples (whole milk, gastric and intestinal digestates) had <10% cytotoxic effect on HEp-2 cells.

**Sample**	**Sample dilution number for** ** <10% cytotoxicity**		
	**Unpurified**	**Experiment 2.2.1** **(Centrifugation twice** **at 11,292 *g* and** **0.2-μm filtration)**	**Experiment 2.2.9** **(Protein G spin column** **and 30-kDa MWCO)**
Whole milk	204.78	8.44	<4
Gastric digestate	505.72	9.26	8
Intestinal digestate	–	–	<4

*Samples (gastric and intestinal digestates) were collected from an infant as described in section Digestion of Human Milk. –: Not determined*.

These findings agree with prior research indicating that components of human milk and digesta, such as proteases, protease inhibitors, and immunoglobulins (SIgA, IgG and IgM), β-casein, lactoferrin and lactoperoxidase, milk fat, cells and bacteria, are capable of inhibiting RSV infectivity (4–7, 12). Many active proteases, including plasmin, elastase, kallikrein and carboxypeptidases are present in human milk (13). As milk moves through the infant digestive tract, numerous proteases are added, including pepsin in the stomach and an array of pancreatic proteases in the intestine. Milk proteases continue to be active in the stomach, as our previous study demonstrated that milk cathepsin D was inactive in human milk but was activated by the low pH environment within the stomach (14). Although the exact relationship of proteolytic activity to viral inhibition is currently unknown, proteases may degrade and neutralize RSV. For example, trypsin from Atlantic cod (*Gadus morhua*) reduced viral titers of RSV via viral degradation (15). Therefore, proteases in the samples used in the present study could interfere with determining the inhibition of RSV infection of HEp-2 cells by palivizumab.

Human milk contains a broad array of protease inhibitors, including α1-antitrypsin, α1-antichymotrypsin, α2-antiplasmin, plasma serine protease inhibitor and antithrombin III (16). Serine protease inhibitors [4-(2-Aminoethyl)benzenesulfonyl fluoride hydrochloride (AEBSF) and N-p-Tosyl-L-phenylalanine chloromethyl ketone (TPCK)] inhibited RSV A2 infection of HEp-2 cells (12). Host cell proteases are necessary for initiating the infection cycle of RSV through specific cleavage of the F protein into F1 and F2 subunits, which is required for the activation of membrane fusion (12, 17). Thus, the presence of protease inhibitors in milk and milk digestive samples could inhibit RSV activity. Moreover, as protease inhibitors are known to impair RSV infection, we could not apply exogenous protease inhibitors to the samples to prevent the proteolytic breakdown of palivizumab without affecting the RSV neutralization assay.

Human milk contains abundant β-casein proteins, which have RSV inhibitory activities (18). In addition, milk contains lactoferrin and lactoperoxidase, which bind to a specific region of the RSV F protein, thereby inhibiting viral adsorption and replication (7, 19, 20). Human milk also contains an array of naturally occurring polyclonal immunoglobulins (IgA, IgG, and IgM), of which some fraction may bind to RSV (5, 21). The presence of these protein components could also interfere with the ability to detect the effect of palivizumab in the sample via the neutralization assay. Similarly, monoglycerides and free fatty acids exhibit RSV antiviral activity (5, 22). The presence of these lipids in the samples could interfere with detecting palivizumab functionality. Various cells or bacteria present in milk or added in the stomach or intestine could also degrade palivizumab or RSV particles or have cytotoxic effects on HEp-2 cells, thus inhibiting our ability to see the effect of palivizumab in the neutralization assay.

The components of human milk and simulated digestates without palivizumab had a direct, significant background effect on observed cytotoxicity and neutralization. These background effects interfered with determination of the additional effect of palivizumab in the RSV neutralization assay. Detection of changes in palivizumab functional capacity across infant digestion was required for our overall study. Therefore, we needed to isolate palivizumab and remove other neutralizing substances prior to the neutralization assay. Numerous potential approaches are available for antibody purification that are based on unique physical and chemical properties such as size, solubility, charge, hydrophobicity and binding affinity, but none had been tested with recombinant antibodies, such as palivizumab, isolated from milk, gastric, and intestinal matrices. Thus, various purification approaches were tested for recovery of palivizumab and removal of interfering substances from milk and infant digestates. The approaches examined were centrifugation, membrane filtration for sterility, MWCO filtration, salting-out of immunoglobulins, affinity chromatography (protein A and G) and dialysis (Experiments 2.2.1–2.2.9, Table 3).

**Table 3:** Palivizumab recovery from human milk and infant digestates with different extraction methods for removal of sample background neutralization effects.

**Expt. No**.	**Purification method**	**Palivizumab recovery, %**		
		**Human milk**	**Gastric**	**Intestinal**
2.2.1	Centrifugation (11,292 *g*) and 0.2-μm filtration	80.57	100.46	–
2.2.2	Centrifugation (4,000 *g*), 100-kDa MWCO and 0.2-μm filtration	83.49	59.32	92.51
2.2.3	Salting out	10.25	–	–
2.2.4	Skimming fat, casein precipitation, 0.2-μm filtration and 100-kDa MWCO	8.76	–	–
2.2.5	Protein A column (Gravity-flow method)	25.15	1.89	–
2.2.6	Protein G spin plate (low-pH elution)	60.13	38.20	73.32
2.2.7	Protein G spin plate (low-pH elution) and 100-kDa MWCO	2.73	2.14	2.94
2.2.8	Protein G spin plate (low-pH elution) and 100-kDa dialysis	22.86	12.94	9.05
2.2.9	Protein G spin column (high-salt elution and 30-kDa MWCO)	64.20	77.25	71.07

–*: Not determined*.

To remove fat, coagulated casein particles, some cells and bacteria from human milk and infant digestates to reduce their potential interfering effect for the neutralization assay, we tested centrifugation. Centrifugation can be used to remove fat and coagulated casein at 4,000 *g* (23) and bacteria at 10,000 *g* (24). We also tested the use of a 0.2-μm filter to remove cells, bacteria and residual casein particles. Extraction of palivizumab after centrifugation (2 times; 11,292 *g*, 10 min at 4°C) and filtration (0.2 μm) was nearly complete for milk and gastric samples based on the palivizumab anti-idiotype ELISA (Table 3; Experiment 2.2.1). These purified samples from human milk and gastric digestate were also tested for cytotoxicity against HEp-2 cells. The centrifuged samples were less cytotoxic than the unpurified samples and exhibited <10% cytotoxicity on HEp-2 cells at any dilution >10 × (Table 2). Despite lower cytotoxicity, the sample had a high background effect in the neutralization assay, likely due to a high level of interfering substances that remained in the sample.

To further remove interfering substances, we applied a 100-kDa MWCO centrifugal filter step to the palivizumab purification approach. As palivizumab has a relatively large molecular weight of 148-kDa (25), it should be retained on MWCO, whereas smaller proteins (including proteases and protease inhibitors) and molecules such as bile salts should be removed via diffusion into the permeate. Palivizumab recovery after centrifugation to remove lipids, 100-kDa MWCO centrifugal filtration and 0.2-μm filtration in milk, gastric and intestinal samples was relatively high (Table 3; Experiment 2.2.2). Each sample with no added palivizumab was extracted in the same way and tested in the plaque reduction neutralization assay. The sample dilution number for insignificant effect of background on the neutralization assay was beyond the maximum number of dilutions tested in the experiment (>864 ×; Table 1). This finding indicates a need for further removal of background substances prior to palivizumab functional testing. The remaining interfering substances may have been components larger than 100-kDa, such as IgA, IgM, or casein micelles.

As another approach for removal of interfering substances, we tested direct isolation of palivizumab and other immunoglobulins in the samples via salting out with ammonium sulfate (Experiment 2.2.3). Immunoglobulins precipitate at 40–50% ammonium sulfate saturation. After salting out, immunoglobulins can be re-solubilized using standard buffers (26). Salting-out of palivizumab from whole milk resulted in a poor recovery of palivizumab (<10% recovery) (Table 3; Experiment 2.2.3). As the recovery with salting-out was poor, we did not test the samples in the neutralization assay.

In another hypothesized method for overcoming the interfering substances, we tested the removal of fat by centrifugation, followed by the elimination of casein—a known inhibitor of RSV infection—via acid precipitation, and 100-kDa MWCO. Proteins are typically least soluble at the isoelectric point, the pH at which the net charge of the protein is neutral. Lowering the pH of samples to the isoelectric point of casein (4.6) destabilizes casein micelles in milk thereby inducing isoelectric precipitation, allowing for casein elimination (27, 28). Palivizumab recovery with this approach was low (Table 3; Experiment 2.2.4). Possibly, palivizumab was lost during the casein precipitation step, perhaps by interacting with the coagulated casein particles.

To improve removal of interfering substances, we tested palivizumab direct extraction via affinity chromatography with a protein A column. Palivizumab, a humanized IgG, consists structurally of two light chains and two gamma heavy chains with fragment antigen binding and fragment crystallizable (Fc) domains (29). Protein A binds with high affinity to the Fc region of IgG (30). Protein A is a protein (42 kDa) found in the cell wall of *Staphylococcus aureus* and contains five Fc binding sites of IgG and the binding occurs optimally at pH 7.5–8.0 (30, 31). Theoretically, palivizumab, an IgG1 subclass antibody, should bind to protein A, allowing washing off of non-binding components followed by antibody elution, enabling purification. We found poor recovery of palivizumab using protein A (Table 3, Experiment 2.2.5). As the approach used included a long extraction time (~5 h), palivizumab could have been degraded by proteases in the sample.

Since protein A affinity chromatography was not an effective method for extraction of palivizumab, we tested a protein G spin plate. Protein G differs from protein A in binding specificity. Protein G is also a bacterial cell wall protein (32 kDa) found in group G *Streptococcus* and contains two IgG Fc region-binding domains and binding occurs optimally at pH 5 (31, 32). Palivizumab recovery efficiency from whole milk and *in vitro*-digested samples (gastric and intestinal samples) treated with this method ranged from 38 to 74% (Table 3, Experiment 2.2.6). The improved recovery compared with protein A could be due to the faster extraction time (~55 min), the differing binding specificity or the differing binding and elution buffers used. We performed a neutralization assay with samples extracted by the same method without added palivizumab. The background effect was reduced compared with the unextracted sample and centrifuged, 100-kDa MWCO filtered and 0.2 μm-filtered sample (Table 1), but it was still too high to detect palivizumab functionality. If we start with feeding human milk with 100 μg/mL palivizumab (our IRB-approved feeding concentration) and assume up to 50% degradation in the infant stomach and intestine, we would have 50 μg/mL remaining in the intestinal samples. If we assume a 50% loss during extraction (based on this method), this would result in 25 μg/mL. We would need to dilute the sample at least 78-fold to eliminate the background effect across sample types. Diluting the sample 78-fold would yield 0.32 μg/mL palivizumab. This concentration is below the typical 50% plaque neutralization concentration of palivizumab in our assays (0.57 μg/mL). Based on these calculations, we would not be able to differentiate the effect of the added palivizumab from the background interference with this method.

To further decrease interfering substances, we applied a 100-kDa MWCO filtration step after the protein G spin plate. This combined procedure resulted in > 97% palivizumab loss (Table 3, Experiment 2.2.7). The buffer used to elute palivizumab from the protein G spin plate (Experiment 2.2.6–2.2.8) had a low pH (pH 2–3). Some studies (33, 34) reported conformational changes in antibodies at low pH leading to aggregation and loss. Thus, a potential acid shock could have made palivizumab more susceptible to further degradation when exposed to the mechanical forces of the MWCO centrifugation step.

We hypothesized that a gentler method for removing interfering compounds that would perhaps allow more time for proteins to refold after acid shock from the protein G spin plate eluant would increase palivizumab extraction efficiency. Therefore, we tested 100-kDa dialysis (18 h). We found that the recovery was better than the protein G and 100-kDa MWCO centrifugal filter unit approach (palivizumab recovery of 9–22%; Table 3, Experiment 2.2.8), but the yield was not high enough for our needs. The relatively low recovery could be due to degradation from milk, gastric and intestinal proteases in the samples during the 18 h of dialysis. Therefore, we hypothesized that a quicker sample purification strategy could increase palivizumab recovery.

To eliminate a potential acid shock, we shifted to a high salt-based elution rather than a low pH-based elution. We also eliminated the possibility of palivizumab activity loss due to pH changes when binding to the column by changing the binding buffer from a neutral pH to a slightly more acidic pH. Having eliminated the potential for acid shock, we selected a rapid centrifugal filtration approach to minimize time for protease interaction by using a MWCO centrifugal filter to remove residual interfering substances. This time, we chose to use a 30-kDa MWCO membrane because using a 100-kDa MWCO could allow some losses of palivizumab in the permeate due to heterogeneity of the pore sizes. Using a 30-kDa filter ensured retention of palivizumab while allowing removal of substances >30 kDa. For this test, palivizumab recovery was 64.20, 77.25, and 71.07% from whole milk, gastric and intestinal digestates, respectively (Table 3, Experiment 2.2.9). This extraction efficiency met our criteria of >50%. These purified samples had <10% cytotoxicity on HEp-2 cells at any dilution above 8 × (Table 2). Additionally, these samples did not significantly affect the background (*p* > 0.05) on the RSV neutralization assay at any dilution above 30 (Table 1). If we feed 100 μg/mL of palivizumab to infants, assume 50% degradation in the gastrointestinal system and 50% loss during extraction, and need to dilute at least 30 times to eliminate the background effect we would have 0.83 μg/mL palivizumab. This concentration is above our typical 50% plaque neutralization concentration of palivizumab in our assays (0.57 μg/mL). Based on this calculation, we would be able to observe the effect of added palivizumab above that of the background at this dilution. However, to generate a reliable PRNT_50_ value (the antibody titer required to reduce RSV plaques by 50 % compared with control), a curve is required with values at higher neutralization as well. The residual background effect at higher palivizumab concentrations (lower dilutions) would likely interfere with the higher PRNT_50_ values, thus limiting the accuracy of our PRNT_50_ calculation. Therefore, we will likely need to increase the palivizumab concentration somewhat in future feeding studies to ensure that we can overcome the residual background effect while maintaining a sufficiently high palivizumab concentration.

Purification of palivizumab using protein G spin columns with a high-salt elution buffer followed by 30-kDa MWCO filtration had the best combination of palivizumab recovery and removal of interfering substances for the viral inhibition assay of the methods tested herein. To verify the feasibility of this extraction approach, mother's milk without or with palivizumab at 60 μg/mL was fed to an infant, and gastric and intestinal contents were collected. The collected samples (milk, gastric and intestinal digestates) were purified using protein G spin columns with a high-salt elution buffer followed by 30-kDa MWCO filtration and the RSV neutralization capacity was determined (Figure 1). We demonstrated that the neutralization effect of palivizumab can be observed in all sample types above the effect of the background. This finding demonstrates that interfering substances were removed effectively while maintaining palivizumab functional activity. Therefore, this approach will enable determination of the functional stability of palivizumab across infant digestion.

## 4. Conclusion

Our overall goal is to examine the survival of recombinant antibodies across the infant digestive system. As a model, we are testing the survival of palivizumab. We found that human milk and infant digesta were intrinsically highly cytotoxic to HEp-2 cells and had a strong RSV neutralizing capacity on their own. Therefore, to detect the functional capacity of remaining palivizumab across digestion, we needed a method to extract palivizumab and remove the interfering neutralizing components of human milk and infant digesta. To achieve this aim, we tested various approaches for antibody purification from human milk, and infant gastric and intestinal samples. Those methods included centrifugation, molecular weight cut-off centrifugal filter units, salting out, acid precipitation, affinity chromatography with protein A and G and dialysis. Of the approaches tested, affinity chromatography with protein G spin columns with a high-salt elution buffer followed by 30-kDa MWCO membrane centrifugal filtration was the most promising to remove inhibitory substances from human milk and infant digestates (gastric, and intestinal) for the RSV neutralization assay while maintaining a high palivizumab extraction efficiency. This approach allowed observation of the neutralization effect of palivizumab above the effect of background in each digestive sample type. This work is broadly applicable to the isolation of antibodies from human milk and digesta and will enable examination of how digestion affects the functional capacity of antibodies within milk and digestates. This work advances our goal to be able to test recombinant antibody survival and function across digestion. Eventually, this work will enable testing of the possibility for the oral administration of enteric pathogen-specific antibodies in the prevention of enteric pathogen-induced infectious diarrhea.

## Data Availability Statement

The raw data supporting the conclusions of this article will be made available by the authors, without undue reservation.

## Ethics Statement

The studies involving human participants were reviewed and approved by Institutional Review Board of Oregon Health & Sciences University (OHSU IRB #18274). Written informed consent to participate in this study was provided by the participants' legal guardian/next of kin.

## Author Contributions

BNPS and JL performed the ELISA. BNPS performed plaque reduction neutralization assay. BNPS, JL, BH, VD-M, BS, MP, and DD designed the study and drafted the manuscript. BNPS and DD had primary responsibility for the final content. All authors contributed to the article and approved the submitted version.

## Conflict of Interest

The authors declare that the research was conducted in the absence of any commercial or financial relationships that could be construed as a potential conflict of interest.

## Abbreviations

DMEM: Dulbecco's Modified Eagle Medium
ELISA: enzyme-linked immunosorbent assay
FBS: fetal bovine serum
MWCO: molecular weight cut-off
PES: polyethersulfone
RSV: respiratory syncytial virus.
